# Sequencing and phylogenetic analysis of the complete mitochondrial genome of the Eurasian Harvest Mouse (*Micromys minutus*) Pallas 1771 from China

**DOI:** 10.1080/23802359.2024.2417974

**Published:** 2024-10-23

**Authors:** Hong-Yan Wang, Yu-Qi Wu, Zhi-Hui Zhang, Zhu Liu

**Affiliations:** College of Life Science and Technology, Mudanjiang Normal University, Mudanjiang, P.R. China

**Keywords:** *Micromys minutus*, mitochondrial genome, phylogenetic analysis

## Abstract

This study aimed to examine the complete mitochondrial genome sequence of the Eurasian Harvest Mouse (*Micromys minutus*) through polymerase chain reaction. The mitochondrial genome of *M. minutus* was characterized as a circular, double-stranded DNA molecule, encompassing a total length of 16,239 bp. The mitochondrial genome composition of *M. minutus* was found to include 13 protein-coding genes, a single control region, 22 tRNA genes, 2 rRNA genes, and a origin of L-strand replication. The accurate identification, sequencing, and assembly of the *M. minutus* mitochondrial genome were successfully conducted in this study, providing a resource for the scientific community to accurately attribute the mitochondrial genome of this species.

## Introduction

*Micromys minutus* Pallas 1771 belongs to the order Rodentia, family Muridae, subfamily Murinae, genus *Micromys* (Wilson and Reeder 2005). Within the *Micromys*, two species have been identified: the Eurasian Harvest Mouse (*Micromys minutus*) and the Indochinese Harvest Mouse (*Micromys erythrotis*) (Abramov et al. 2009). The complete mitochondrial genome of *M. minutus* (KP399599) was reported (Jing et al. 2015). However, we found that the mitochondrial genome (KP399599) that had been reported was not that of *M. minutus*, but that of *Micromys erythrotis*. The similarity between the *CYTB* gene sequence of the mitochondrial genome (KP399599) and that of the reported *Micromys erythrotis* (OQ576199) is 99.12% (Chen et al. 2023). In this study, we sequenced the complete mitochondrial genome of real *M. minutus* and conducted a phylogenetic analysis to elucidate the intrafamilial relationships within Murinae. Our results provide new molecular evidence that contributes to a clearer understanding of the phylogenetic structure within the subfamily Murinae.

## Materials and methods

**Figure 1.**
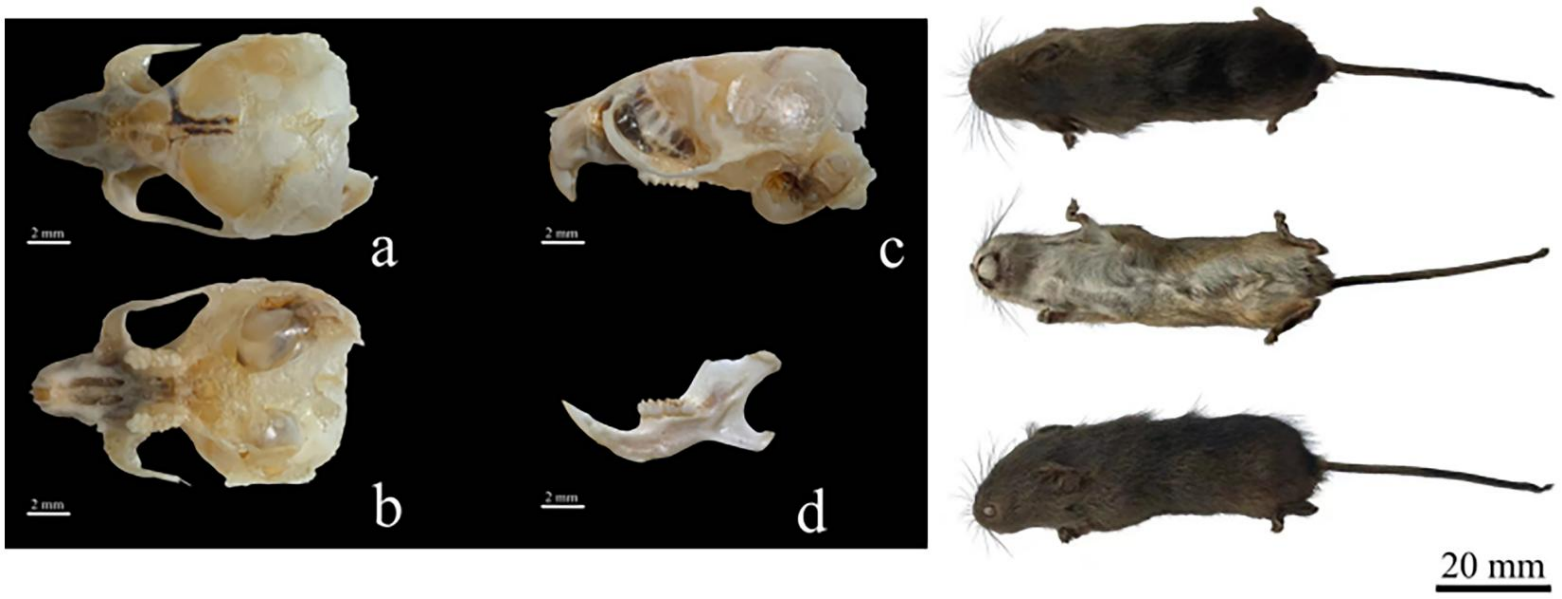
Pictures of external and skull morphologies. Pictures courtesy of Liu Zhu. a: Dorsal view of the maxilla; b: Ventral view of the maxilla; c: Lateral view of the maxilla; d: Lateral view of the mandible. Sequencing specimen is shown on the right.

A male *M. minutus* sample was collected from Hengdaohezi City (44°48′44″N, 129°02′04″E), Heilongjiang Province, China, in September 2023 (Figure 1). The species identification was based on morphological characters as described (Chen et al. 2023) and *CYTB* gene sequence comparison. The *CYTB* gene sequence of the specimen was blasted against the GenBank database. The sequence AB125099 was the most similar to the *CYTB* gene sequence the specimen from *M. minutus* (Yasuda et al. 2005), with an E-value of 0.0, a percentage of identity of 99.21%, and a query coverage of 99%. The sample was stored at −75 °C before use and deposited at the Animal and Plant Herbarium of Mudanjiang Normal University (Liu Zhu, swxlz0@126.com) under the voucher number 2023HD80. Genomic DNA was extracted from leg muscle using the EasyPure genomic DNA kit (TransGen Biotech Co., Beijing, China). We designed 17 pairs of primers for polymerase chain reaction (PCR) based on the reported mitochondrial genome of *Micromys* (Figure 1). The first-generation sequencing technology was used for sequencing in this study (ABI 3730 sequencer; Boshi Biotechnology Co. Ltd., Haerbin, China). The sequences were assembled using DNAstar, analyzed, and adjusted manually. The annotation of the *M. minutus* mitochondrial genome was performed using web-based services MITOS (Bernt et al. 2013) and software PhyloSuite v 1.2.2 (Zhang et al. 2020). The circular mitochondrial genome map of *M. minutus* was drawn using SnapGene 6.0.2 (Tello et al. 2022). A molecular phylogenetic study of *Micromys* was conducted, wherein complete mitochondrial genomes from 10 species of two closely related genera within Murinae (*Rattus* and *Niviventer*) were selected using the Genbank database (Jing et al. 2015). The phylogenetic tree was constructed using 13 protein-coding genes of the complete mitochondrial genome. The software MEGA 11.0 (Tamura et al. 2021) was used to align the sequences, find the best model of maximum likelihood, and construct a phylogenetic tree using the maximum likelihood method with 1000 bootstrap replicates.

## Results

**Figure 2.**
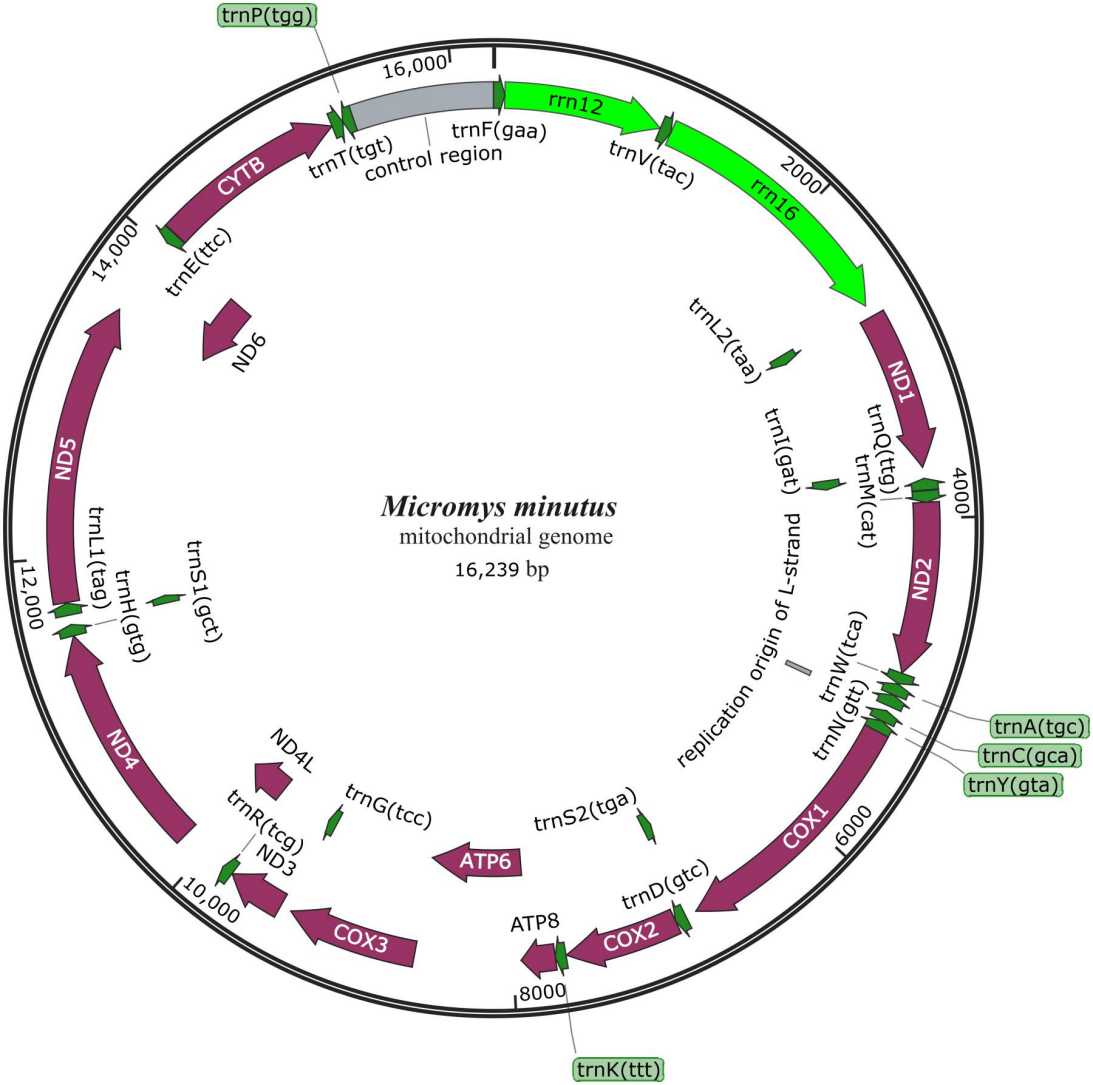
Circular mitochondrial genome map of *M. minutus.* Clockwise represents its position in the H chain, counterclockwise means it’s in the L chain. Green, rRNA; violet, protein-coding gene; gray, replication origin of L-strand and control region.

**Figure 3.**
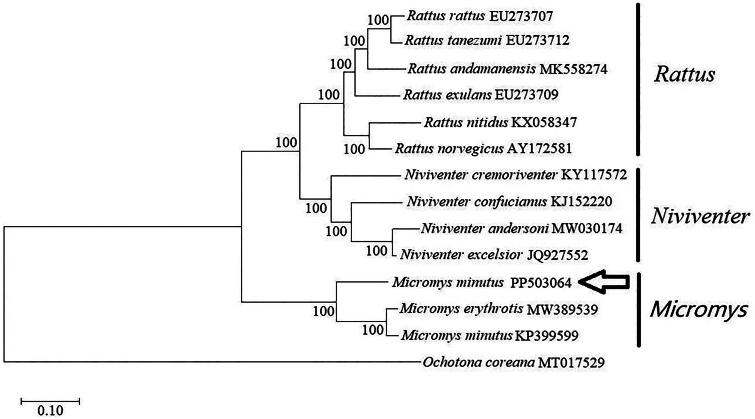
Phylogenetic tree was constructed using 13 protein-coding genes of the complete mitochondrial genome through MEGA 11.0 software, and also constructed using the kimura 2-parameter model of maximum likelihood method with 1000 bootstrap replications: *Rattus rattus*, *Rattus tanezumi*, *Rattus andamanensis*, *Rattus exulans*, *Rattus nitidus*, *Rattus norvegicus*, *Niviventer cremoriventer*, *Niviventer confucianus*, *Niviventer andersoni*, *Niviventer excelsior* (Chen et al. 2012), *Micromys minutus* (PP503064), *Micromys erythrotis* (Cai et al. 2021), *Micromys minutus* (Jing et al. 2015). The outgroup was *Ochotona coreana* (Zhang et al. 2020).

A circular double-stranded structure made up the mitochondrial genome of *M. minutus* (Figure 2). The length of the complete mitochondrial genome is 16, 239 bp. The mitochondrial genome of *M. minutus* includes 13 protein-coding genes, one control region, 22 tRNA genes, 2 rRNA genes, and one origin of L-strand replication (Figure 2). The base composition of *M. minutus* mitochondrial genome is A (33.64%), T (28.70%), G (12.32%), and C (25.34%). Base composition was AT-biased, particularly within the control region and protein-coding genes. The *ND6* gene and eight tRNA genes of *M. minutus* are encoded on the L strand. The other mitochondrial genes are encoded on the H strand (Figure 2). The control region of the mitochondrial genome exists between the *trnP* and *tenF* (Figure 2). The total length of 13 protein-coding gene sequences is 11,373 bp. The lengths of 22 tRNA genes are between 59 and 75 bp. The length of L-strand replication origin (*OL*) is 34 bp. The phylogenetic tree (Figure 3) showed that the three genera (*Niviventer*, *Rattus* and *Micromys*) in the Murinae subfamily each formed independent branches. *M. minutus* and *M. erythrotis*. formed independent branches (Figure 3). *Micromys* was supported by the bootstrap values of 100% (Figure 3). Our results suggest a sister relationship between *M. minutus* and *M. erythrotis* within the phylogenetic tree (Figure 3).

## Discussion and conclusions

The phylogenetic analysis, revealed that the three genera, *Niviventer*, *Rattus*, and *Micromys*, within the Murinae subfamily, each constituted distinct clades on the evolutionary tree. the *M. minutus* with the accession number KP399599 shows a high level of genetic similarity to *M. erythrotis* indicating a close kinship. Conversely, the *M. minutus* PP503064 is divergent from KP399599 on the phylogenetic tree, suggesting significant genetic differences. This supports the reclassification of KP399599 is *M. erythrotis*. The findings of this study will provide a widely accepted reference standard, ensuring that the mitochondrial genomes of *M. minutus* and *M. erythrotis* are accurately classified and assigned. Furthermore, this research will lay a solid foundation for subsequent studies in the fields of evolutionary biology, molecular systematics, and biodiversity conservation.

## Data Availability

The genome sequence data that support the findings of this study are openly available in GenBank of NCBI at [https://www.ncbi.nlm.nih.gov] (https://www.ncbi.nlm.nih.gov/) under the accession no. PP503064. The associated **BioProject**, **SRA**, and **Bio-Sample** numbers are PRJNA1102980, SRR29259702 and SRR29660012, and SAMN41038287 respectively.
